# USP10 deletion inhibits macrophage-derived foam cell formation and cellular-oxidized low density lipoprotein uptake by promoting the degradation of CD36

**DOI:** 10.18632/aging.104003

**Published:** 2020-11-10

**Authors:** Xiaohong Xia, Tumei Hu, Jinchan He, Qiong Xu, Cuifu Yu, Xiaolin Liu, Zhenlong Shao, Yuning Liao, Hongbiao Huang, Ningning Liu

**Affiliations:** 1 Guangzhou Institute of Cardiovascular Disease, Guangdong Key Laboratory of Vascular Diseases, State Key Laboratory of Respiratory Disease, The Second Affiliated Hospital, Guangzhou Medical University, Guangzhou, Guangdong, China; 2 Guangzhou Municipal and Guangdong Provincial Key Laboratory of Protein Modification and Degradation, School of Basic Medical Sciences, Guangzhou Medical University, Guangzhou, China

**Keywords:** atherosclerosis, foam cell, CD36, USP10, degradation

## Abstract

Foam cell formation process is involved in the pathogenesis of atherosclerosis (AS). Activation of this biological process depends on lipid uptake by scavenger receptors, such as CD36, SR-A and SR-B1. Among these receptors, CD36 is the principal one because it dominates roughly 50% lipid uptake in monocytes. In this study, our western blotting and RT-qPCR assays revealed that USP10 inhibition promotes the degradation of CD36 protein but does not change its mRNA level. In addition, Co-IP results showed that USP10 interacts with CD36 and stabilizes CD36 protein by cleaving poly-ubiquitin on CD36. Significantly, USP10 promotes foam cell formation. Immunofluorescence and Oil red O staining assays show that inhibition or knockdown of USP10 suppresses lipid uptake and foam cell formation by macrophages. In conclusion, USP10 promotes the development and progression of atherosclerosis through stabilizing CD36 protein expression. The regulation of USP10-CD36 may provide a significant therapeutic scheme in atherosclerosis.

## INTRODUCTION

Cerebrovascular and cardiovascular diseases are the main causes of incidence and mortality all over the world. Atherosclerosis (AS) is regarded as a most important driver, characterized by infiltration of macrophages in the aorta and uncontrolled lipid metabolism [1]. Evidence now supports that macrophage foam cell is a critical element of AS lesions and promotes the development of AS [2, 3]. Firstly, macrophages are formed by the migrates of monocytes to intima for differentiation. Macrophages metabolism and phagocytosis of oxidized low-density liporotein (oxLDL) are upregulated and then the transported factors bring lipid from cells to the vessel walls. The foam cells are developed to promote progression of AS once the oxLDL intake is more than the capacity of macrophage metabolism. Growing reports demonstrated that the important early step is foam cell formation in the development of atherosclerosis [4–8]. So controling foam cell formation is significant in the inhibition of AS.

OxLDL intake plays a vital process in atherosclerotic plaque formation. In this progression of macrophages-uptake oxLDL needs some factors, including scavenger receptor-A (SR-A), lectin-like oxLDL receptor 1 (Lox-1), CD36 [9, 10]. Not only lipid uptake, but also cholesterol efflux can induce foam cell formation. The process is regulated by ATP-binding cassette transporters A1 and G1 (ABCA1 and ABCG1) and scavenger receptor class B type 1 (SR-B1) [11, 12]. CD36 is a most driver in the formation of foam cells among these factors. Blocking CD36 expression using antibody can induce 50% inhibition of monocyte-derived macrophages and reduce the capacity of oxLDL binding [13, 14]. The stimulation of oxLDL triggers protein expression of CD36 to promote foam cell formation. In apo E^-/-^ mice of genetic deletion of CD36, foam cell formation is decreased. More and more studies explore the regulation of CD36 to mediate the development of AS [15, 16]. Protein expression involves in function. It has been reported that CD36 expression can be degraded by ubiquitin proteasome system (UPS) [17].

UPS consists of E1, E2, E3, 26S and deubiquitinases (DUBs). DUBs are well known enzymes that regulate the ubiquitin cycle through cleaving the ubiquitin chains from their substrates [18]. DUBs play vital role in the cellular processes [19]. Ubiquitin-specific peptidase 10 (USP10) belongs to mammalian deubiquitinases. It has been reported that USP10 mediates cancer progression via stabilizing some substrates, such as p53, STRT6, AMPK, FLT3 and androgen receptor [20–24]. In our previous study, we have reported that USP10 stabilizes skp2 to promote the development of CML [25]. But the pathophysiology of USP10 in atherosclerosis has not been validated.

In this study, we sought to demonstrated that USP10 inhibition induced downregulation of lipid accumulation and foam cell formation. Moreover, CD36 expression is stabilized by USP10 through cleaving ubiquitin on CD36. These findings may represent a therapeutic scheme of cardiovascular disease.

## RESULTS

### USP10 mediates lipid uptake by macrophage

**Figure 1:**
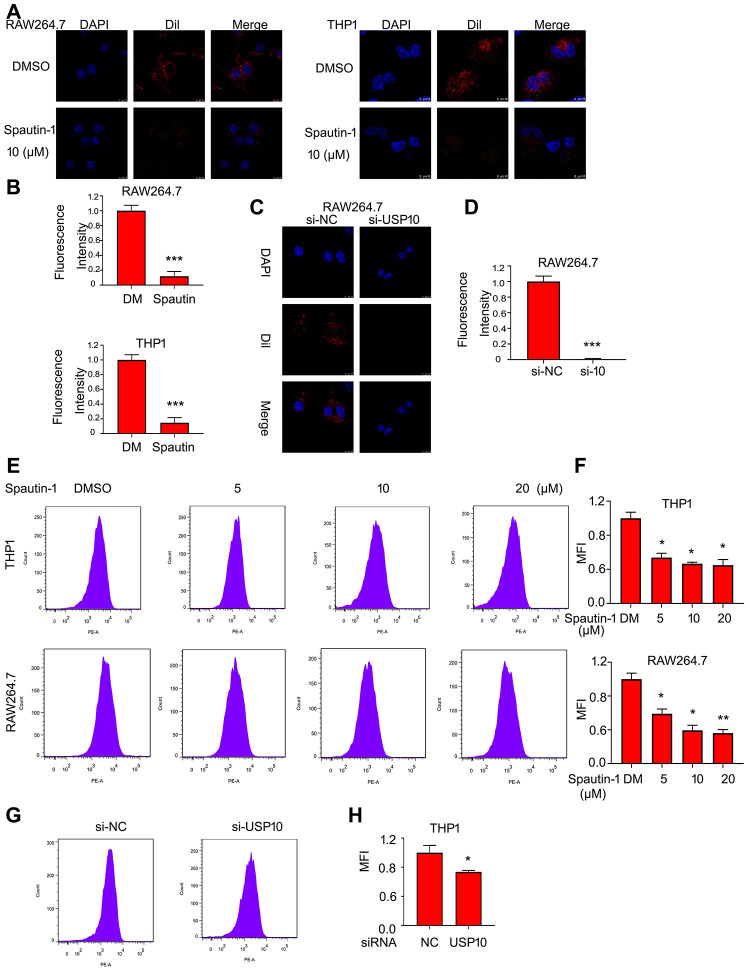
**USP10 mediates lipid uptake by macrophage.** Cells were treated with Spautin-1 for 24 h or USP10 siRNA for 48 h. Dil-oxLDL was added for the additional 6 h. DAPI for cell nucleus. The images were taken by confocal microscopy (**A**, **C**) and by flow cytometry (**E**, **G**). The fluorescence intensity was performed using Image pro plus (**B**, **D**) and quantitative analysis was showed (**F**, **H**). *p<0.05, **p<0.01, ***p<0.001 *versus* each vehicle control. DM:DMSO.

It has been reported that lipid intake is the first step in development of AS. Lipid is ingested by macrophage differentiated from monocyte. To detect the role of USP10 in lipid intake by macrophage, we used the oxLDL labeled by fluorescent dye Dil. The confocal assay demonstrated that USP10 inhibition induced fluorescent intensity, indicating that USP10 inhibitor (Spautin-1) reduced lipid uptake by THP1 and RAW264.7 cells (Figure 1A, 1B). In addition to inhibitor, we applied siRNA to further explore the function of USP10. Delightfully, USP10 deletion significantly decreased the lipid uptake in RAW264.7 cells (Figure 1C, 1D). Moreover, we also applied flow cytometry assay to demonstrate the ability of USP10 for lipid uptake. We found that Spautin-1 remarkably inhibited oxLDL uptake by macrophages (Figure 1E, 1F). The USP10 siRNA also blocked THP1 cells to intake oxLDL labeled by fluorescent dye Dil (Figure 1G, 1H).

### USP10 inhibition or deletion diminishes the formation of foam cell

**Figure 2:**
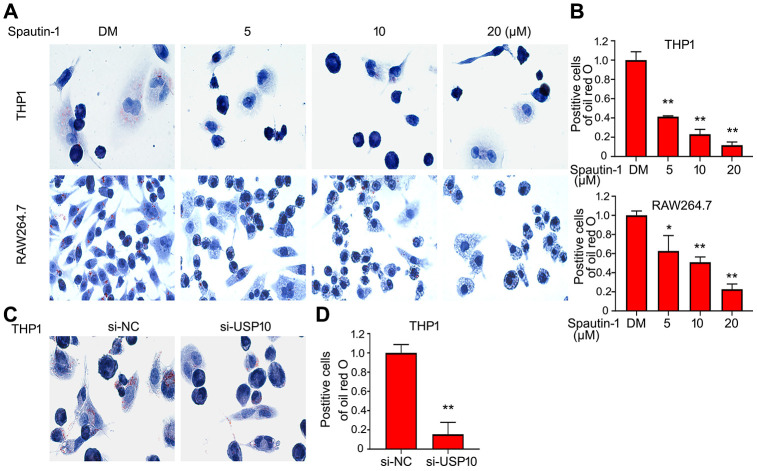
**USP10 inhibition or deletion diminishes the formation of foam cell.** (**A**, **C**) Cells were treated with Spautin-1 or USP10 siRNA and oxLDL (50 μg/ml) for the indicated time. Cell was stained with oil red o. The represent images were showed from three independent experiments. (**B**, **D**) The number of stained cells were counted. *p<0.05, **p<0.01 *versus* each vehicle control.

Given that USP10 inhibitor or siRNA significantly reduced Dil-oxLDL uptake by macrophages, we speculated whether foam cell formation is influenced by USP10. Firstly, we used oxLDL to incubate cell in THP1 and RAW264.7 cells. Oil red O assay showed that stained cell number was decreased in the treatment of USP10 inhibitor, suggesting that inhibition of USP10 blocks the foam cell formation in THP1 and RAW264.7 cells (Figure 2A, 2B). What is more, USP10 siRNA was applied. As shown in Figure 2C, 2D, knockdown of USP10 induced the same results using oil red O assay. These results demonstrated that USP10 involved in the formation of foam cell.

### USP10 regulates the protein expression of CD36

**Figure 3:**
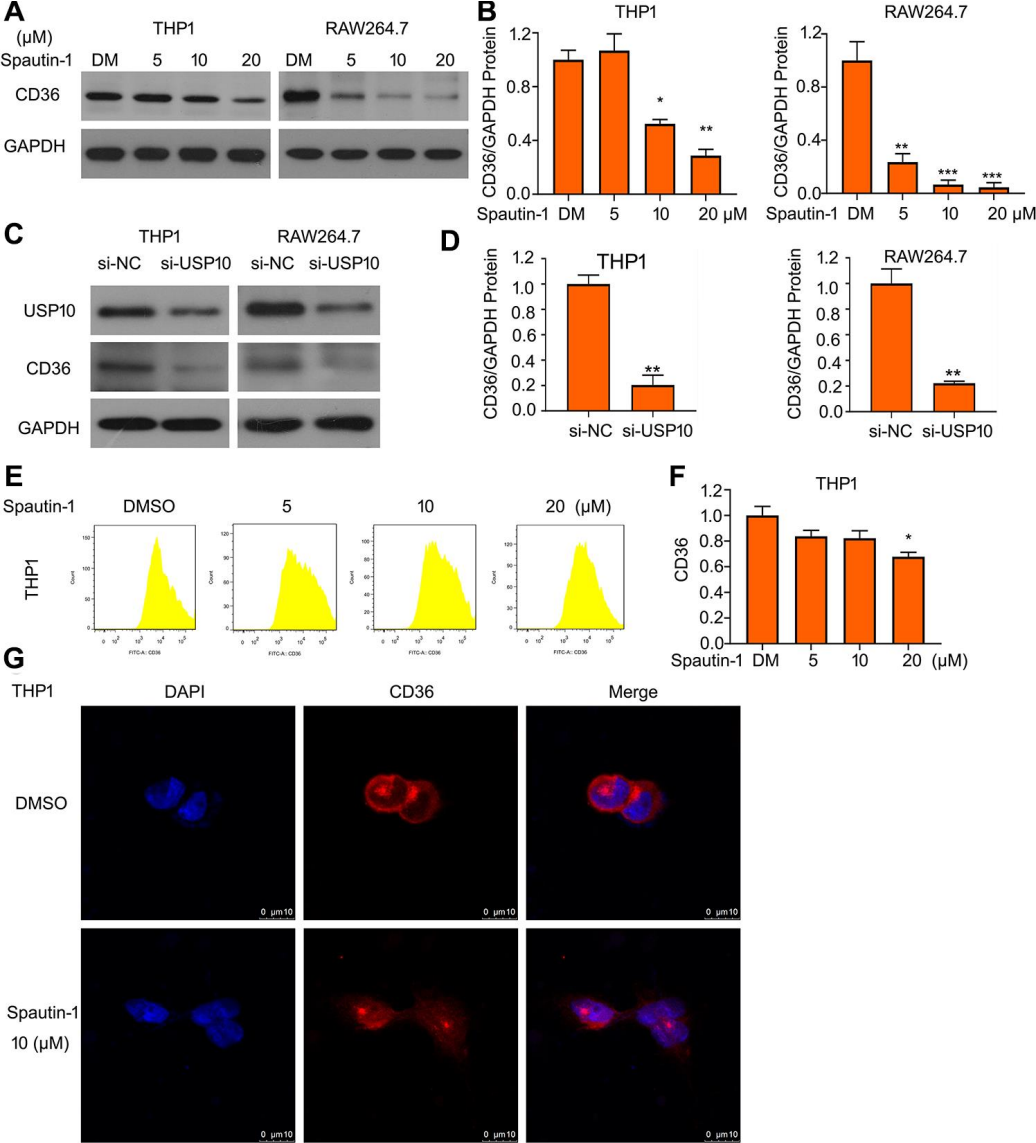
**USP10 regulates the protein expression of CD36.** (**A**, **C**) THP1 and RAW264.7 cells were exposed to Spautin-1 or USP10 siRNA. Protein was extracted and subjected to western blot for expression of CD36. (**B**, **D**) The quantitation of CD36 band were performed. (**E**, **F**) The treated cell posted with Spautin-1 was stained with FITC-labeled CD36 antibody followed by flow cytometry. (**G**) Cells were stained with CD36 and Cy3-conjugated antibody and subjected to confocal microscopy. *p<0.05, **p<0.01, ***p<0.001 *versus* each vehicle control.

CD36 represents a vital function in progression of AS through mediating lipid intake by macrophage. We have clarified that USP10 inhibition suppresses lipid uptake. To further explore the regulatory mechanism of USP10, we tested the molecular expression under treatment of USP10. Interestingly, western blotting showed that Spautin-1 downregulated the protein expression of CD36 in THP1 and RAW264.7 cells (Figure 3A, 3B). USP10 knockdown also inhibited the expression of CD36 protein (Figure 3C, 3D). To further conformed USP10 induced-lipid uptake just depends on CD36, we evaluated other scavengers expressions (SR-B1, SR-A, Lox-1, ABCA1 and ABCG1). Western blot assay was used and the results showed that protein expressions are not regulated by UP10 inhibitor and siRNA (Supplementary Figure 1A, 1B). To ensure the effect of USP10 on CD36 expression, we used FITC-labeled CD36 antibody to perform flow cytometry assay. We found that USP10 inhibitor significantly reduced CD36 expression (Figure 3E, 3F). Moreover, we further observed CD36 location after the treatment of USP10 inhibitor. The confocal assay showed that CD36 is a membrane protein and USP10 changed the expression but not affect the translocation of CD36 protein (Figure 3G).

### OxLDL-induced the upregulation of CD36 is reduced by USP10 inhibition

**Figure 4:**
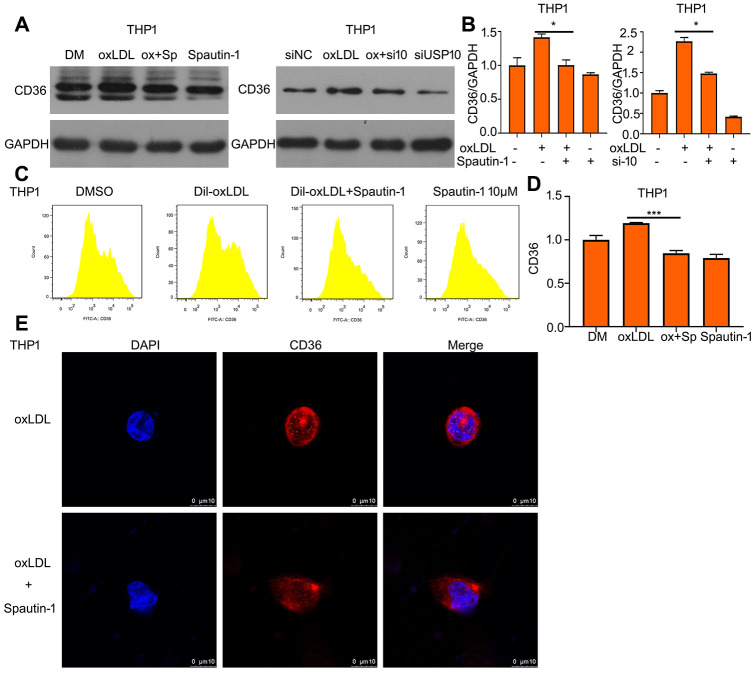
**OxLDL-induced the upregulation of CD36 is reduced by USP10 inhibition.** (**A**) Cells were treated with Spautin-1/USP10 siRNA, oxLDL or the combination of the two treatments. Protein was harvested for western blot assay to test CD36 expression. (**B**) The band of CD36 was counted. (**C**, **D**) The treated cells posted with Spautin-1 were stained FITC-CD36 antibody followed by flow cytometry. (**E**) THP1 cell was incubated with CD36 antibody and DAPI for cell nucleus. *p<0.05, ***p<0.001 *versus* each vehicle control.

As known, it is a positive loop between oxLDL and CD36 expression. Lipid uptake can be upregulated by increasing CD36 expression and CD36 expression can be increased by oxLDL stimulation. Therefore, we assessed whether USP10 also regulated the protein expression of CD36 under oxLDL treatment. Western blot showed that increased expression of CD36 protein was suppressed by USP10 inhibitor or siRNA (Figure 4A, 4B). Using FITC-labeled CD36 antibody demonstrated that Spautin-1 significantly reduced oxLDL-induced increased CD36 expression (Figure 4C, 4D). CD36 location also was observed after the oxLDL + Spautin-1. We found that USP10 inhibition decreased the expression of CD36 in THP1 cells (Figure 4E).

### USP10 stabilizes CD36

**Figure 5:**
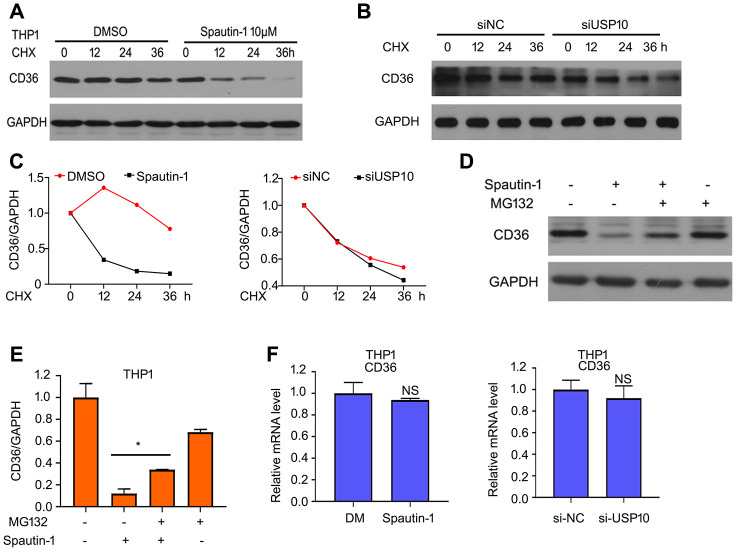
**USP10 stabilizes CD36.** (**A**, **B**) THP1 cells were treated with CHX or CHX+Spautin-1/USP10 siRNA for 0, 12, 24 and 36 h. CD36 expression was detected using western blot assay. (**C**) And then the band of CD36 was calculated. (**D**, **E**) Cells were treated with Spautin-1, MG132. Western blot was performed to test the expression of CD36. (**F**) Total RNA was extracted and RT-qPCR was performed to detected mRNA level of CD36. *p<0.05 *versus* Spautin-1 treatment.

To better understand the role of USP10 on expression of CD36 protein, we sought to explore how USP10 stabilizes CD36 expression. We speculate whether the regulation is happen in the transcription level. Firstly, we used cycloheximide (CHX) to block protein synthesis. We found that the half-life period is shorter in the treatment of Spautin-1/USP10 siRNA and CHX than in the treatment of CHX in THP1 cells (Figure 5A–5C). Considering that CD36 is degraded by UPS, we used MG132 which blocking 20S proteasome activity to observe the protein expression of CD36. Western blot assay showed that blocking 20S proteasome activity abrogated the inhibition of CD36 expression induced by Spautin-1 (Figure 5D, 5E). In addition to transcription level, we also explore the translation level. The result of RT-qPCR showed that mRNA level of CD36 has no effect by USP10 inhibition or knockdown (Figure 5F). The results suggested that USP10 regulated CD36 expression via transcription method rather than translation method.

### USP10 interacts with CD36

**Figure 6:**
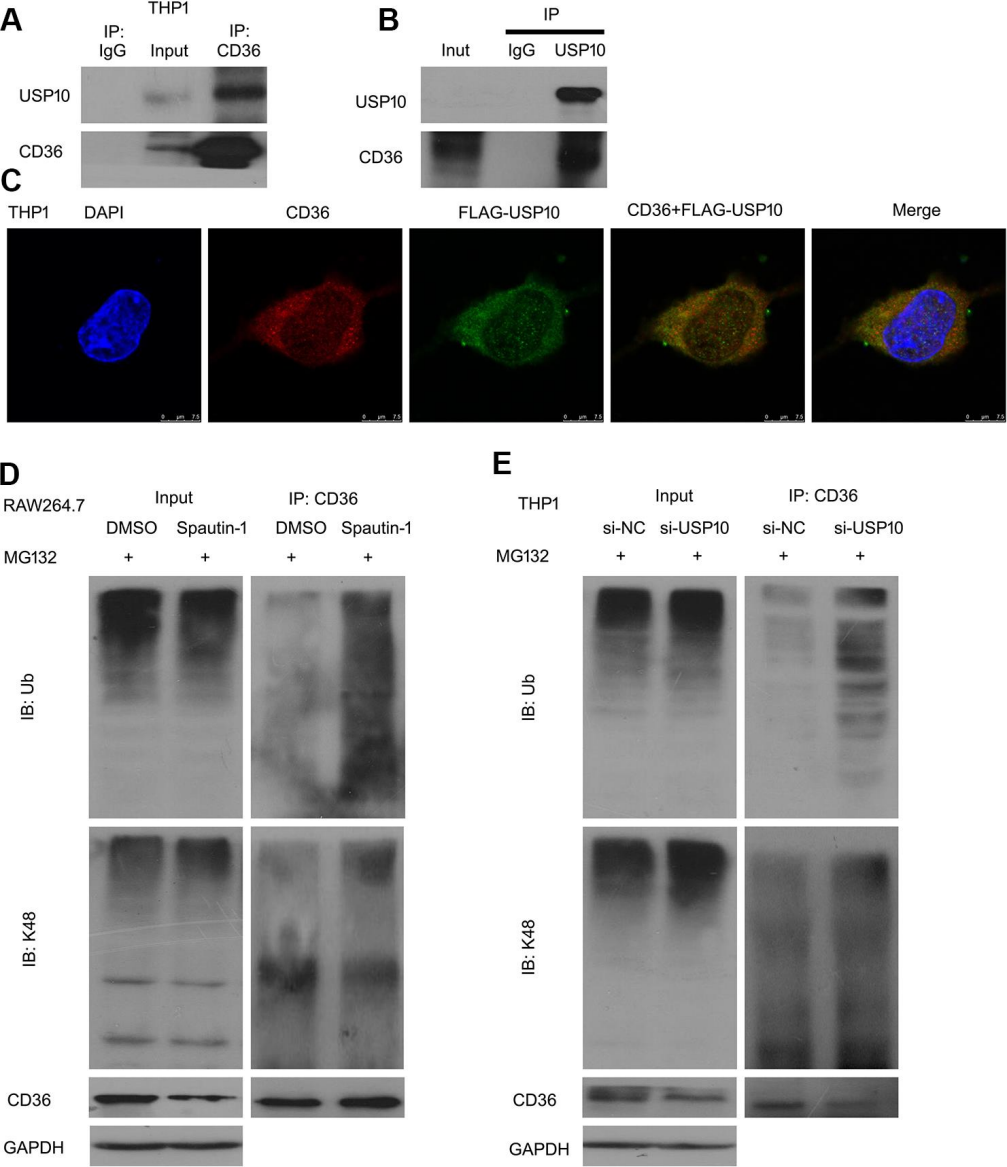
**USP10 interacts with CD36.** (**A**, **B**) Protein lysates were extracted from THP1 cells and subjected to Co-IP assay. (**C**) Cells were transfected with FLAG-tagged USP10 for 48 h and then stained with FLAG and CD36 antibodies, followed by confocal assay. (**D**, **E**) Cells were treated with Spautin-1 or USP10 siRNA. Immunoprecipitated with CD36 beads was subjected to immunoblotted for Ub and K48.

Multiple studies have reported that DUBs promote the degradation of their substrate proteins [26]. In this study, we have clarified that USP10 is involved in CD36 protein stability. Then we speculated that CD36 is substrate protein of USP10. We co-immunoprecipitation (Co-IP) with antibodies against CD36 or USP10 followed by immunoblot (IB) with antibodies against USP10 or CD36. Western blot results showed that USP10 interacts with CD36 (Figure 6A, 6B). Moreover, we observed the cellular location of CD36 and USP10 using confocal assay. Cells were transfected with FLAG-USP10 (green) and we found that FLAG-tagged USP10 appeared to be co-localized with endogenous CD36 (Figure 6C). Next, we hypothesized that USP10 stabilizes CD36 via deubiquitination. To investigated the effect, we used Co-IP assay to test ubiquitinated CD36. As shown in Figure 6D, 6E, USP10 inhibition or knockdown significantly increased ubiquitinated CD36. Moreover, the K48-poly-ubiquitinated CD36 was upregulated by Spautin-1 or USP10 siRNA.

### USP10-mediated lipid uptake depends on CD36 expression

**Figure 7:**
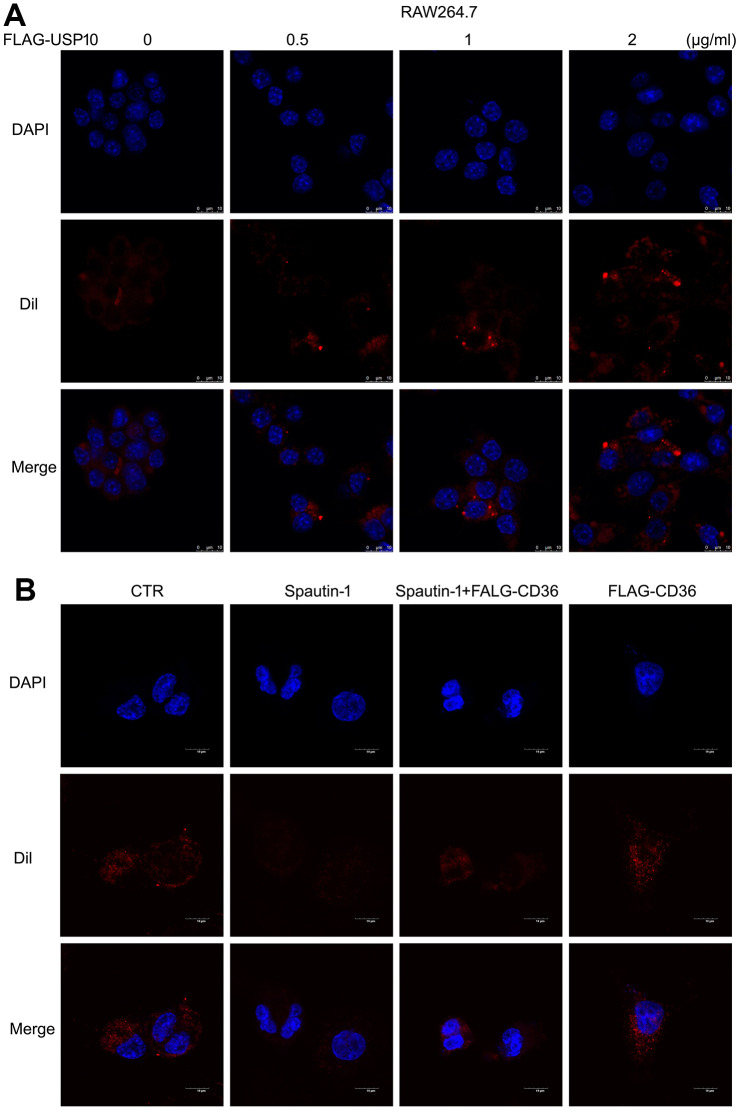
**USP10-mediated lipid uptake depends on CD36 expression.** (**A**) Macrophage was transfected with FLAG-USP10 plasmid with different doses for 48 h, followed by Dil-oxLDL for 6 h. (**B**) Macrophage was treated with Spautin-1 and /or FLAG-CD36 plasmid for 48 h. Dil-oxLDL was added for the additional 6 h. DAPI for cell nucleus. The images were taken by confocal microscopy.

We have studied what silencing USP10 induced the inhibition of lipid uptake and CD36 expression. To further verify whether USP10 mediates lipid uptake by macrophage, we overexpressed USP10 to evaluate lipid uptake using Dil-oxLDL. Conversely, the result of fluorescence intensity showed that overexpression of USP10 increased lipid uptake (Figure 7A). Moreover, given that USP10 regulated CD36 expression, we determine if re-introduction of CD36 would rescue the lipid uptake inhibition induced by USP10 inhibitor. To do so, THP1 cells were transfected with FLAG-CD36 plasmid or a control vector, followed by Spautin-1. Overexpression of CD36 was able to rescue USP10 inhibition-induced lipid intake (Figure 7B). These results suggested that CD36 is indispensable for USP10-meidiated lipid uptake by macrophage.

## DISCUSSION

Atherosclerosis (AS) is a metabolic disorder accompanied by inflammation including adaptive immune and innate cells [27, 28]. A known pathological progression is that macrophages intake excessive amount of lipids resulting in foam cell formation. Forming foam cell is a hallmark of the early stages of AS involving in the formation of fatty streak. Blocking foam cell formation could be a plan for therapeutic target [29]. AS reaction from foam cell is generated by macrophages which are rooted in blood-borne and smooth muscle cells monocytes [30, 31]. *In vitro*, 50 μg/ml oxLDL for 24 h helps macrophages evolve typical foam cells via two aspects which are lipid intake and cholesterol afflux. Both pathways, some molecules play main role for uptake and transport, including in CD36, SR-A, Lox-1, ABCG1, SR-B1 and ABCA1 [32]. Among these, CD36 is regarded as the most important receptor in foam cell formation. Compelling studies evidence that CD36 and its downstream signaling pathways in macrophages show crucial roles in AS [33, 34]. Even though numerous evidences show the role of CD36 in the formation of foam cell, more detailed molecular regulation mechanism that participate in this progression of AS and the critical process of foam cell formation is still necessary [35].

The protein stabilize of CD36 depends on ubiquitin proteasome system (UPS) which includes the 19S, 20S and deubiquitinases (DUBs). DUBs takes charge of recognizing and removing ubiquitin moieties from their substrates [19]. In our previous study, we firstly reported that a DUB of CD36 is USP14 [36]. Significantly, we found that USP10 regulates the expression of CD36 protein. USP10 belongs to mammalian DUBs. USP10 is well documented involving in the progression and development of several cancers. USP10 regulates different substrates, such as AMPK, p53, SKP2 and androgen receptor in cancer [20, 21, 24, 25]. To date, the role of USP10 in AS remains unknown.

In the current study, our primary objective is to explored function of USP10 in oxLDL uptake by macrophages. The results show that the inhibition or silence of USP10 significantly induce the decreased of lipid intake. What is more, the formation of foam cell is inhibited by USP10 inhibitor or siRNA in RAW264.7 and THP1 cells. To further know the regulation mechanism, we speculated if scavenger receptors is related in the process of USP10-induced foam cell formation. Western blotting analysis demonstrated that the protein expression of CD36 was suppressed by inhibiting or silencing USP10. Flow cytometry and confocal assay showed that USP10 only regulated protein expression of CD36 in cytomembrane rather than its translocation. Besides, we tested the regulation of USP10-CD36 under the oxLDL stimulation. OxLDL treatment induced the upregulation of CD36 protein. Importantly, USP10 deletion suppressed the increased of CD36 induced by oxLDL. Moreover, USP10 inhibition or silence did not trigger other receptors increase or decrease, including in ABCA1, ABCG1, Lox-1, SR-A, SR-B1.

Deeply, we sought to the regulation mechanism of CD36-USP10 in macrophages. Transcription and translation levels are contribute to protein expression. In transcription aspect, we explore the mRNA level of CD36. USP10 did not change the mRNA level of CD36. Then in translation level, we found that USP10 inhibitor promoted the degradation of CD36 and the MG132 which blocking 20S rescued the protein expression of CD36. Based on this, we assessed that USP10 regulated CD36 via UPS. Co-IP and confocal assays revealed that CD36 interacts USP10 and the co-localization of CD36 and USP10. Under treatment of USP10 deletion, the poly- and poly-K48 ubiquitin on CD36 were increased, suggesting USP10 stabilizes CD36 protein by removing its ubiquitin.

**Figure 8:**
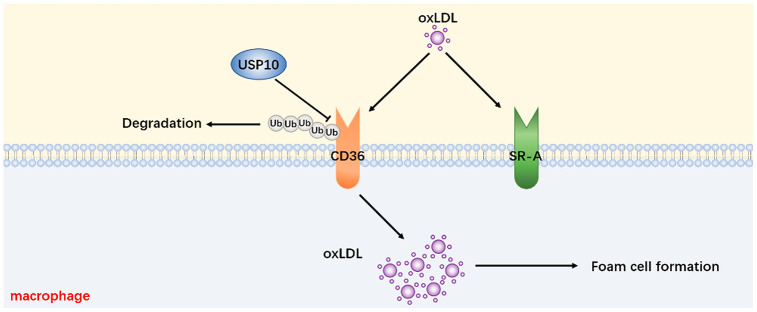
**A proposed mechanism for USP10 to regulate foam cell formation via stabilizing the CD36 level.**

In summary, we have uncovered the function of USP10 on foam cell formation via regulating CD36 protein degradation (Figure 8). The present study also has investigated that USP10 is a DUB of CD36 and as a potentially practicable scheme for AS.

## MATERIALS AND METHODS

### Materials

Spautin-1 and MG132 were purchased from Selleckchem (Houston, TX, USA). Control (SC-37007) and USP10 (SC-76811) siRNAs were from Santa Cruz Biotechnology (Santa Cruz, CA, USA). Human dil-labeled oxLDL (YB-0010) oxidized low density lipoprotein (oxLDL) (YB-002) were from Yiyuan Biotechnologies (Guangzhou, China). Antibodies are as follows: anti-GAPDH (#5174), anti-USP10 (#8501), anti-ubiquitin (#3936), anti-K48-linkage Specific Polyubiquitin (#12805), anti-CD36 (ab13365), ABCA1 (#96292), ABCG1 (ab52617), SR-B1 (ab217318), SR-A (ab123946). Lox-1 was from R&D system. Oil Red O was from Sigma-Aldrich. CO-IP were obtained from Novus biologicals (USA).

### Cell lines and culture conditions

THP1 cell from human and RAW264.7 cell from murine were purchased from ATCC (Manassaa, VA, USA). THP1 cells were cultured by RPMI 1640 with 10% FBS and stimulated by PMA (200 ng/ml). And RAW264.7 cells were cultured by DMEM ((Thermo Fisher Scientific)) with 10% FBS.

### Oil red O staining

Cells were treated with USP10 inhibitor or siRNA for the indicated time. OxLDL was used to incubated with additional 24 h. Cell was fixed and washed with PBS, followed with Oil red O which dissolved with 0.5% in isopropyl alcohol for 3-5 min. Then 60% isopropanol and water were applied to wash cell. Cell nucleus was stained with hematoxylin and washed by water. Images were captured by optical microscope.

### Dil-oxLDL uptake

Cell was treated with Spautin-1 or USP10 siRNA in confocal cuvette for the indicated time. OxLDL labeled by fluorescence was applied to incubated with cell for 6 h. Cell was fixed and washed followed by cell nucleus staining with DAPI. Images were captured and fluorescence intensity was counted by Image Pro Plus software.

### Western blot and Co-IP

The assay was performed as we previously reported [37, 38]. Total protein was harvested in cell treated with the indicated treatment. For Co-IP assay, in our previous studies [25], protein was reacted with the mixture of antibodies and dynabeads for 1-2 h. Then the reaction was washed with PBS-T for three times and loading buffer was applied to suspended. Lastly, protein was subjected to SDS-PAGE followed by transferring to membrane. The membrane was incubated with antibodies including primary antibody and second antibody.

### Plasmids and siRNA transfection

The assay was performed as same in our previously study [39]. The plasmid was purchased from Genechem (Shanghai, China). Cell was plated into dishes and cultured with the mixture of plasmid and lipofectamine 3000 reagent (Life Technologies). For siRNA transfection, cell was incubated with the mixture of iMAX and USP10 siRNA for 48 h.

### Flow cytometry

The treated cells were stained by fluorescence-labeled oxLDL or FITC-labeled CD36 antibody. Cell was washed with PBS for two times, followed by flow Cytometry analysis. fluorescence intensity was counted.

### Immunofluorescence

The immunofluorescence assay was performed as we reported [40]. Cell was plated into chamber slide and exposed to the indicated treatment. 0.1% Triton-X 100 dissolved with PBS was applied to react with cell and then 5% BSA was used to block for 30 min. Cell was incubated with primary antibody for one night at 4 °C and secondary antibody for 1 h. DAPI was used for cell nucleus. Images were taken using confocal microscope.

### RT-qPCR

The assay was performed as same in our previous study [41]. The total RNA was extracted. And then the purity and concentration of RNA were evaluated at 260:280 nm. CDNA was synthesized using an equal amount of RNAs. mRNA was measured using real-time quantitative PCR. The primes are as following: CD36: forward: 5-TTTCCTCTGACATTTGCAGGTCTA-3 and reverse: 5-AAAGGCATTGGCTGGAAGA-3; GAPDH: forward:5-ACCCAGAAGACTGTGGATGG-3 and reverse: 5-ACACATTGGGGGTAGGAACA3.

### Statistical analysis

The represent data are as mean±SD. Analyzing the statistical significance of differences using unpaired Student's t test. All statistical analyses were performed using SPSS 22.0 and GraphPad Prism 8.0. The level of p < 0.05 was regarded to be significant.

## Supplementary Material

Supplementary Figure 1
