# Supplementary material for: USP10 deletion inhibits macrophage-derived foam cell formation and cellular-oxidized low density lipoprotein uptake by promoting the degradation of CD36
Source: Aging (Albany NY). 2020 Nov 10;12(22):22892–905. doi: 10.18632/aging.104003 (PMC7746336; doi:10.18632/aging.104003)
Supplement: Supplementary Figure 1 [file aging-12-104003-s001..pdf]

SUPPLEMENTARY FIGURE

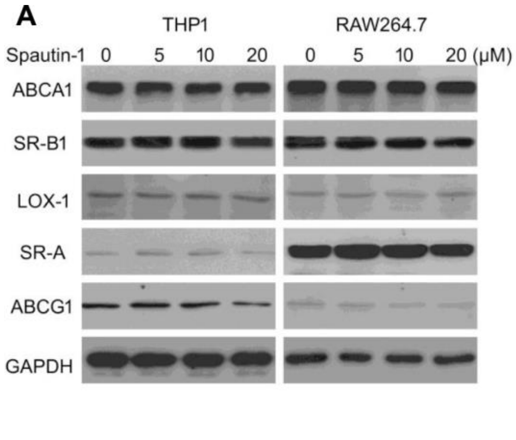

Supplementary Figure 1. USP10 mediates lipid uptake by macrophage. (**A**) Cells were treated with Spautin-1. Western blot assay was employed for ABCA1, ABCG1, SR-A, SR-B1 and Lox-1 protein expressions. (**B**) THP1 cell was exposed to USP10 siRNA and then test proteins expressions, including in ABCA1, ABCG1, SR-A, SR-B1 and Lox-1 protein expressions.
